# Heatwave interventions must reduce invisible gendered challenges in the Global South

**DOI:** 10.1371/journal.pgph.0003625

**Published:** 2024-10-24

**Authors:** Haiwei Li, Ronita Bardhan, Ramit Debnath

**Affiliations:** University of Cambridge, Cambridge, United Kingdom; PLOS: Public Library of Science, UNITED STATES OF AMERICA

## Introduction

Heat-health risks in the Global South are becoming increasingly high due to climate change, urbanization, and socio-economic disparities. Rising temperatures exacerbate health vulnerabilities, particularly among densely built areas and marginalized communities with limited access to cooling infrastructure and healthcare resources. These compounding hazards pose a threat to public health and strain already fragile healthcare systems in the vulnerable communities of the Global South [1].

According to the World Health Organization, heatwaves caused over 166,000 deaths worldwide between 1998 and 2017, with women suffering more than men [2]. For instance, a recent estimate from Europe suggests that women experienced approximately 56% more heat-related deaths than men between May 30 and September 4, 2022 [2]. However, such gender-specific attributable numbers remain unclear for the Global South, posing a significant challenge for heatwave intervention design [3]. Many countries do not systematically count health-related deaths, and some may actively suppress such information. For example, India has faced challenges for underreporting heat-related mortality. A lack of reliable data makes it difficult to assess the full extent of heat-related health risks, let alone understand gender-specific risks with reliable statistics [3, 4].

**Fig 1:**
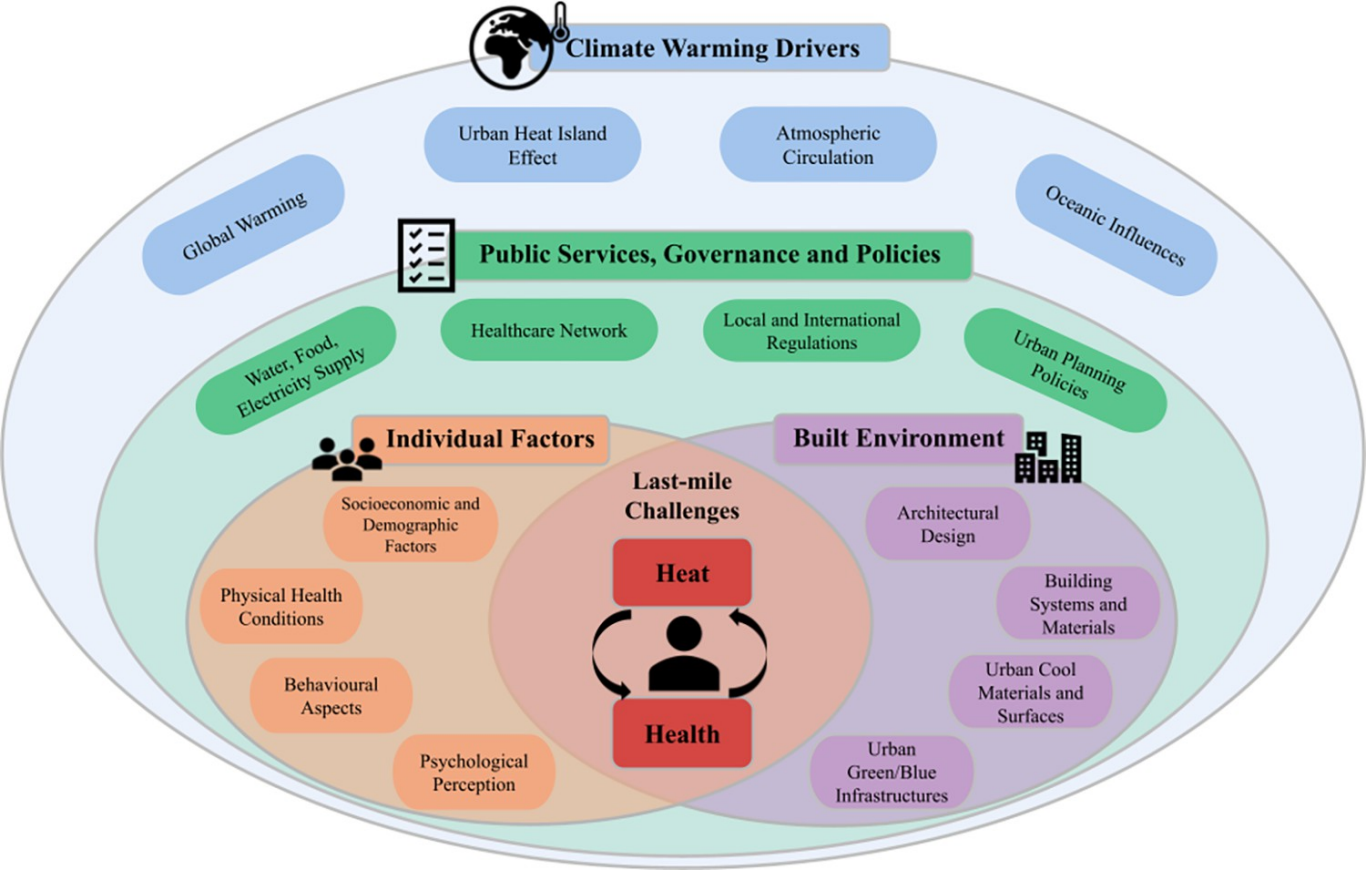
Interconnected factors impacting heat-related health in the Global South. This figure illustrates the components of climatic drivers, public services, governance and policies, individual factors, and the built environment. The interwoven factors of individuals and the built environment directly connect to the challenges for heat mitigation.

Understanding the interconnected factors that contribute to heat-related health risks is crucial for ensuring effective community-level heatwave interventions. As illustrated in Fig 1, these factors include climate change drivers, public services and policies, built environment characteristics, and individual factors such as socio-economic status, demographic attributes, psychological conditions, pre-existing health issues, human behavior, and access to public services. These interconnected factors constitute ‘invisible urban infrastructures’ that are not immediately visible or tangible but play a crucial role in shaping daily life, well-being, and resilience. They are social, cultural, and economic systems that influence the accessibility and affordability of cooling services, shape urban sustainability, and contribute to a community’s overall climate resilience [5, 6].

Here, we focus on the role of invisible infrastructures, and their connection to heat-health in vulnerable communities in the Global South. It explores potential actions for designing context-specific interventions in resource-constrained settings that ensures community’s resilience to environmental and social challenges.

## Addressing gendered heat-health challenges in the Global South

Heatwaves disproportionately affect women in the Global South. A recent study based on 30 years of gender-stratified mortality data in India highlighted that women are more vulnerable to high temperatures than men [3]. The Asian Development Bank estimates that in Asia and the Pacific region, there is a starling gender gap in access to cooling, with an estimated 60% more women lacking access compared to men [7]. Factors such as poverty traps, poor built environment design, and socio-cultural norms further exacerbate these vulnerabilities [6]. Addressing these challenges is therefore critical for designing mitigation strategies and avoiding maladaptation to heat-related health risks.

Due to the deep interconnections between individual factors, urban and building infrastructures, and gender-specific heat-health hazards, adaptation and mitigation plans for heatwaves in the Global South must untangle these complex issues [8]. This includes reducing or eliminating indoor and outdoor heat sources that increase vulnerabilities, such as the lack of shading, green spaces, or well-ventilated areas. Poorly planned urban areas and housing designs hinder cross-ventilation, limiting natural airflow in indoor spaces, which exacerbates risk factors for women [9, 10]. For instance, women often bear the responsibility of cooking and fetching water, which exposes them to hot stoves, indoor air pollution, and poorly ventilated kitchens. These spaces frequently lack efficient heat sources and cooling systems, significantly increasing women’s exposure to heat. Moreover, these challenges are particularly severe for women in low-income communities, where limited access to healthcare services and cooling infrastructure, gender disparities in social and economic status, cultural norms that prioritize men’s health, lower health literacy among women, and systemic biases in healthcare services intensify the risks [4, 8, 10].

Therefore, tailored heatwave interventions must consider the unique vulnerabilities faced by women in the Global South, ensuring equitable “invisible infrastructures” to mitigate the compounding risks posed by heatwaves.

## Invisible interventions for beating the lethal heat

We envision invisible infrastructure as a system that seamlessly integrates into people’s lives without requiring them to change their individual behavior. Rather than forcing adaptation, this type of infrastructure aligns with social norms and enhances the existing ecosystem. For example, the placement of windows can significantly reduce heat stress through ventilation. However, if windows are installed without considering the community’s need for privacy, they may remain closed, leading to maladaptation [7].

Invisible infrastructure operates in the background, providing essential services and benefits to many people, often unnoticed and underappreciated. These subtle yet impactful interventions are crucial for addressing heat-health challenges in resource-constrained communities, particularly for women. By offering solutions that empower individuals without disrupting existing social practices, invisible infrastructure improves quality of life and provides agency [11, 12]. As a result, invisible infrastructures are essential systems and strategies that, while not directly visible, significantly contribute to adaptation, resilience, and sustainability. These systems provide multiple unseen benefits to the most vulnerable individuals and the built environment [5, 9]. When designing heatwave adaptation plans, it is vital to identify and implement these infrastructures in a context-sensitive manner.

At an urban scale, invisible infrastructure designed with a gendered perspective can serve as passive cooling measures that effectively mitigate the urban heat island effect and improve heat resilience. Examples include urban green spaces, water surfaces, reflective pavements, and community shading zones. In low-income neighborhoods, developing a safe and reliable water supply system can significantly reduce the risks faced by women who would otherwise have to walk long distances in extreme heat to fetch water. Access to electricity can improve living conditions by enabling the use of cooling appliances, such as fans, particularly in homes and kitchens, thereby reducing the harmful effects of cooking heat. Additionally, interventions like clean cookstoves can enhance indoor air quality, reducing health risks associated with smoke inhalation, such as respiratory illnesses. During severe heatwaves, establishing specialized heat-related illness units that provide relief through IV hydration and other emergency treatments can ensure effective care.

Addressing the invisible infrastructural challenges posed by heatwaves in the Global South requires a multifaceted, systematic approach that unravels the complex interplay between public services, governance policies, and human and built environment factors. Persistent data gaps, knowledge deficits, and equity issues related to gender-specific mortality and various social outcomes remain significant challenges in the Global South. Future research should focus on interventions that explore cost-effective, context-specific, and gender-equitable cooling solutions to combat the invisible lethal heat and protect the most vulnerable populations from this growing threat.
